# Artificial intelligence method based on multi-feature fusion for automatic macular edema (ME) classification on spectral-domain optical coherence tomography (SD-OCT) images

**DOI:** 10.3389/fnins.2023.1097291

**Published:** 2023-01-30

**Authors:** Fan Gan, Fei-Peng Wu, Yu-Lin Zhong

**Affiliations:** ^1^Medical College of Nanchang University, Nanchang, China; ^2^Department of Ophthalmology, Jiangxi Provincial People’s Hospital, The First Affiliated Hospital of Nanchang Medical College, Nanchang, China

**Keywords:** artificial intelligence, macular edema, multi-feature fusion, SD-OCT images, classification models

## Abstract

**Purpose:**

A common ocular manifestation, macular edema (ME) is the primary cause of visual deterioration. In this study, an artificial intelligence method based on multi-feature fusion was introduced to enable automatic ME classification on spectral-domain optical coherence tomography (SD-OCT) images, to provide a convenient method of clinical diagnosis.

**Methods:**

First, 1,213 two-dimensional (2D) cross-sectional OCT images of ME were collected from the Jiangxi Provincial People’s Hospital between 2016 and 2021. According to OCT reports of senior ophthalmologists, there were 300 images with diabetic (DME), 303 images with age-related macular degeneration (AMD), 304 images with retinal-vein occlusion (RVO), and 306 images with central serous chorioretinopathy (CSC). Then, traditional omics features of the images were extracted based on the first-order statistics, shape, size, and texture. After extraction by the alexnet, inception_v3, resnet34, and vgg13 models and selected by dimensionality reduction using principal components analysis (PCA), the deep-learning features were fused. Next, the gradient-weighted class-activation map (Grad-CAM) was used to visualize the-deep-learning process. Finally, the fusion features set, which was fused from the traditional omics features and the deep-fusion features, was used to establish the final classification models. The performance of the final models was evaluated by accuracy, confusion matrix, and the receiver operating characteristic (ROC) curve.

**Results:**

Compared with other classification models, the performance of the support vector machine (SVM) model was best, with an accuracy of 93.8%. The area under curves AUC of micro- and macro-averages were 99%, and the AUC of the AMD, DME, RVO, and CSC groups were 100, 99, 98, and 100%, respectively.

**Conclusion:**

The artificial intelligence model in this study could be used to classify DME, AME, RVO, and CSC accurately from SD-OCT images.

## Introduction

Macular edema (ME) is a common ocular manifestation of fluid infiltration or inflammation in the sensitive macular area of the retina, and is an important cause of visual deterioration (Song et al., 2022). There are several ME-related eye diseases, including diabetic ME (DME), retinal-vein occlusion (RVO), age-related macular degeneration (AMD), and central serous chorioretinopathy (CSC). Chronic hyperglycemia in diabetes mellitus (DM) causes damage to capillaries, resulting in retinal ischemia and increased vascular permeability, which leads to DME (Kim et al., 2020). Wet AMD is a result of subretinal choroidal neovascularization, resulting in fragile and leaky blood vessels that penetrate through Bruch’s membrane and cause edema (McGill et al., 2017). Excessive angiogenic growth factors, caused by hypoxia secondary to RVO, leads to vascular leakage and ME (Narayanan et al., 2021). In CSC, choroidal congestion, thickening, and hyperpermeability are considered to cause leakage through the retinal pigment epithelium (RPE) (Schellevis et al., 2019).

Optical coherence tomography (OCT) is a high-resolution, non-contact, and non-invasive biomedical imaging technique and is often used in eye clinics for macular disease. The basic principle of OCT imaging is that a beam of light is emitted into the tissues to be examined and detects the reflected or back-scattered light from the tissues; this reflected light will interfere with light that originated from the same source and the reflectivity profile along the light beam can be derived from the interference signal and used to generate an A-scan (Liu et al., 2019). The combination of multiple A-scans along the horizontal axis produces a brightness scan (B-scan) (Khalid et al., 2017). Compared with Fundus cameras, OCT systems has the high contrast and depth sectioning capability (LaRocca et al., 2014). And high-quality cross-sectional images of the neurosensory retina can be acquired without pupil dilatation in a matter of seconds (Ouyang et al., 2013). Therefore the sensitivity of OCT for detection of a variety of retinal irregularities was higher.

Macular edema diagnosis by OCT is based on the visualization of the retinal structure. However, spectral-domain optical coherence tomography (SD-OCT) can better delineate the different retinal layers so that the histological changes of ME can be shown in more detail. In DME patients, SD-OCT shows mild retinal edema with cystic spaces located only in the outer plexiform layer (OPL), whereas, when edema worsens, they involved both the OPL and the outer nuclear layer (ONL) (Leung et al., 2008). SD-OCT image analysis was also more sensitive than FAF for identifying geographic atrophy GA in patients treated for exudative AMD (Massamba et al., 2019). For CSC patients, SD-OCT can show shallow serous detachments and provided precise information about the amount and localization of subretinal fluid and RPE abnormalities (Murthy et al., 2016). SD-OCT also can quantify retinal thickness changes in eyes with cystoid macular edema (CME) from central retinal vein occlusion (CRVO) and is superior to contact lens–assisted biomicroscopy to identify foveal edema (Decroos et al., 2013).

Currently, ME diagnosis depends on the subjective evaluation of OCT and the clinical experience of ophthalmologists. Not only does this process take a lot of time, energy, and requires training, but the ability of ophthalmologists at different levels to diagnose diseases ranges widely. With the application of artificial intelligence in ophthalmology, a large number of machine learning-based computer-aided diagnosis (CAD) models have been developed for the quantitative analysis of OCT images to achieve the automatic diagnosis of macular diseases. Alsaih et al. (2017) applied machine-learning techniques for DME classification on SD-OCT images, both the sensitivity (SE) and specificity (SP) of the best result were 87.5%. Chen Y. et al. (2021) applied convolutional-neural-network-based transfer learning to classify AMD, the CNN models with appropriate algorithm hyperparameters had excellent capability and performance in classifying OCT images of AMD and DME. However, their studies made only binary classification, which limits the application of machine-learning algorithms in the diagnosis of many diseases. Wang et al. (2016) proposed a CAD model to discriminate AMD, DME, and healthy macula on OCT images, the best model based on the sequential minimal optimization (SMO) algorithm achieved 99.3% in the overall accuracy for the three classes of samples. However, the coverage of disease types was still inadequate and their studies were all based on single features.

**FIGURE 1:**
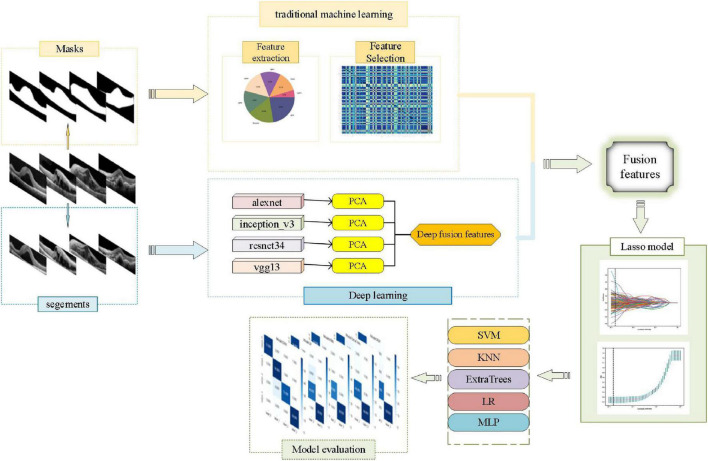
The flowchart of this study.

Currently, the signal fusion methods have attracted the attention of many researchers for solving pattern recognition problems, and that were divided into three categories which are early fusion, intermediate fusion, and late fusion (Verma and Tiwary, 2014). Early fusion is also named as feature level fusion which emphasizes the data combination before the classification (Zhang et al., 2017). It was defined as performing merge and splitting operations on existing feature sets to generate new feature sets. Using the feature fusion approach of deep learning and machine learning, the complementary information of abstract features of deep learning and detailed features of machine learning can be realized (Wang et al., 2022). The accuracy of models could be improved (Khan and Hasan, 2020). Therefore, we introduced an artificial intelligence method of fusion of traditional features and deep features, aiming to automate the classification of DME, AME, RVO, and CSC from DM based on SD-OCT images (Figure 1).

## Materials and methods

### Image collection and pre-processing

A total of 1,213 two-dimensional (2D) cross-sectional OCT images of ME were collected from the Jiangxi Provincial People’s Hospital (China) between 2016 and 2021. According to OCT reports of a senior ophthalmologist, 300 images with DME, 303 images with AMD, 304 images with RVO, and 306 images with CSC were included. And the set was randomly divided into a training set and a test set, at a ratio of 8:2. To protect patient privacy, patient images were all anonymized prior to analysis. All the OCT images were acquired by the same experienced ophthalmologist on the same machine, i.e., Heidelberg Spectralis OCT (Heidelberg Engineering, Dossenheim, Germany). Then, the macular area was outlined manually with ITK-SNAP software, which was the mask region of interest (ROI) images. The obtained mask files were used for traditional omics features extraction. Next, the image was cropped to the ROI specifications and the segments of ROI were used for DL features extraction.

### Traditional omics features extraction and selection

Based on the mask files of the original image files, traditional omics features of images were extracted based on the first-order statistics, shape, size, and texture. The *Z*-score standardization method was then used to normalize the extracted features and the Spearman correlation coefficients were used to select the normalized feature.

### DL features extraction and model visualization

The segments of ROI were input into alexnet, inception_v3, resnet34, and vgg13 models, respectively, which were initialized using the pre-trained weights from ImageNet, and the DL features were obtained. And then the DL features were selected by dimensionality reduction using principal components analysis (PCA). Finally, the selected features of the four DL models were fused.

In order to evaluate the deep learning-focused regions, the gradient-weighted class activation map (Grad-CAM) method was used. In this method, gradient information flowing from input layers to the last convolution layer of a convolutional neural network (CNN) is used, and coarse heat maps of important regions in the input images are generated (Chen T. et al., 2021). Based on the coarse heat maps, we can understand which areas of the segments are most likely to be focused by the DL models.

### Early fusion and lasso model established

Feature fusion was performed after the pooling layer in the model. The traditional omics features and the deep-fusion features were fused into a composite feature vector. Then in the training set, the composite feature vector was input into further fused as fusion features set. A *t*-distributed stochastic neighbor embedding (*t*-SNE) algorithm was used to visualize the features vectors from feature space of high dimensions into 2D space. Then, the fusion features set was divided into a training set and a test set, at a ratio of 7:3. The lasso model which was established to further select features. We chosed the optimal λ based on the minimum criteria according to fivefold cross validation.

### Classification models established

The support vector machine (SVM), K-nearest neighbor (KNN), ExtraTrees, logistic regression (LR), and multilayer perceptron (MLP) were used to establish the classification models in the training set and the performance of the final classification models was evaluated in the test set. Finally, the classification performance of the different models was assessed and compared.

### Statistical analysis

The accuracy, confusion matrix and the receiver operating characteristic (ROC) curve of the classification models were used to evaluate the performance of models. All statistical analyses were performed and visualized in Python (version 3.9.7).

## Results

### Characteristics of OCT images

The total of 1,213 original images of ME were collected, included DME (*n* = 300), AMD (*n* = 303), RVO (*n* = 304), and CSC (*n* = 306). The training set was consisted of 849 images, included DME (*n* = 240), AMD (*n* = 243), RVO (*n* = 243), and CSC (*n* = 245). The test set was consisted of 364 images, included DME (*n* = 60), AMD (*n* = 60), RVO (*n* = 61), and CSC (*n* = 61). And then, the original image files and corresponding mask files were obtained to use for traditional omics features extraction. The segments based on maximum ROI mask were used for deep learning features extraction.

### Characteristics of traditional omics features

**FIGURE 2:**
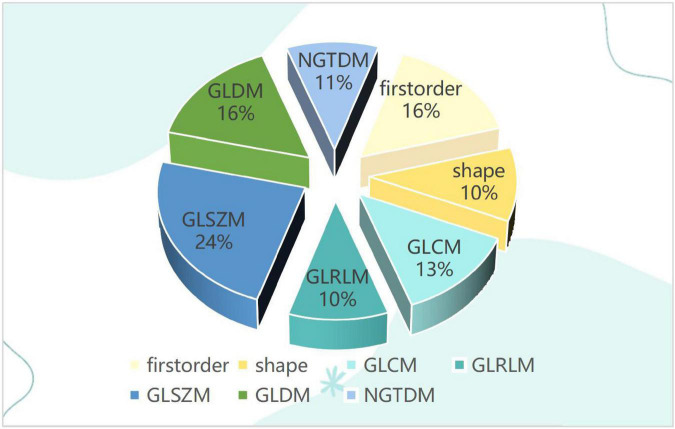
The pie chart for traditional omics features distribution.

For each ROI, a total of 107 features of each image were extracted, and after the Spearman correlation coefficients, the final 38 features of each image were selected. Including 6 first-order features, 4 shape-based features, and 28 textural features. The textural features were composed of 5 Gray Level Co-occurrence Matrix (GLCM), 4 Gray Level Run Length Matrix (GLRLM), 9 Gray Level Size Zone Matrix (GLSZM), 6 Gray Level Dependence Matrix (GLDM), and 4 Neighboring Gray Tone Difference Matrix (NGTDM) as shown in Figure 2.

### Characteristics of deep learning features and model visualization

**FIGURE 3:**
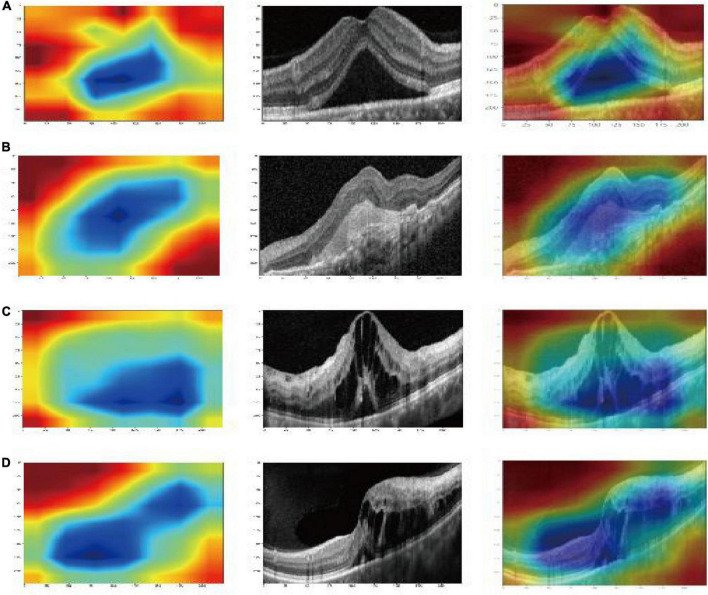
Gradient-weighted class-activation map (Grad-CAM) visualization of deep learning feature extraction: CSC **(A)**; AMD **(B)**; DME **(C)**; RVO **(D)**. The blue part that gathers inward from the red part is active, indicating that the model pays particular attention to this area (Huang et al., 2022).

There were 9,216, 2,048, 512, and 16,383 deep learning features of each image were obtained from the alxnet, inception_v3, resnet34, and vgg13, respectively, which were on “avgpool” layer before last FC layers. Dimension reduction with PCA compressed features into 31. Finally, a deep fusion feature subset containing 124 compression features were obtained. And the heatmaps of Grad-CAM highlighted areas which the deep learning models likely focused on as shown in Figure 3.

### Characteristics of fusion features

**FIGURE 4:**
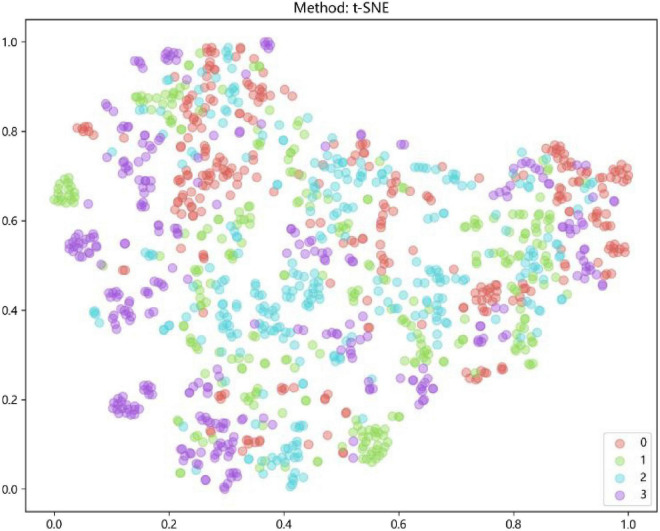
Feature visualization by *t*-distributed stochastic neighbor embedding (*t*-SNE): AMD (red); DME (green); RVO (blue); CSC (purple).

After the early fusion, a subset of each image that contains 162 features was got. Feature was visualized by *t*-SNE for an intuitive perception of how well these features can distinguish different types of ME, was shown in Figure 4.

### Lasso model evaluation

**FIGURE 5:**
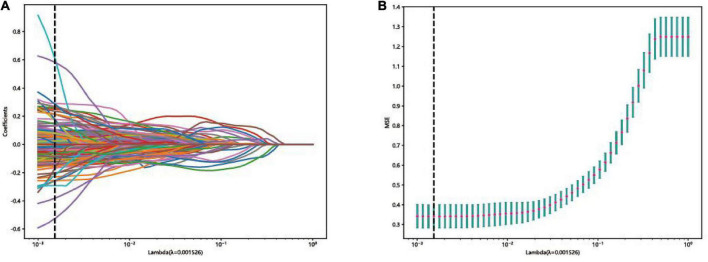
Feature selection in the lasso model: **(A)** Lasso coefficient profiles of the 162 fusion features, where each curve corresponds to one feature, the vertical black line indicates an optimal λ. **(B)** Curve of binomial deviation varied with parameter λ, where value of the optimal log (λ) is marked by vertical dashed lines.

The LASSO was used for automated feature selection in this study. 53 features were selected to build the final classification models based on the optimal lambda value and the corresponding coefficients in the training set as shown in Figure 5.

### Classification models evaluation

The accuracy of SVM model was highest than other models, up to 93.8% in the test set. And the KNN, ExtraTrees, MLP, and LR models in the test set were only 90.08, 82.23, 90.50, and 86.77%, respectivily, as shown in Table 1.

**TABLE 1:** The accuracy of classification models in the training set and test set.

Model	Accuracy	Task
SVM	97.22%	train
	93.80%	test
KNN	90.32%	train
	90.08%	test
ExtraTrees	100%	train
	82.23%	test
MLP	98.35%	train
	90.50%	test
LR	90.83%	train
	86.77%	test

Since our problem is a multiclass classification, AUC of binary class classification cannot be considered. So, micro- and macro-averages (Sokolova and Lapalme, 2009) were calculated from ROC curves, the macro-average could give equal weight to the classification of each label, whereas the micro-average incorporates the frequency of the labels into the label weighting. In the test set, the area under curves (AUCs) of micro- and macro-averages of the SVM and MLP models both were 99%, which was highest than the other models. The ROC curve of the test set for each group compared with that of the other groups, each group were clearly distinguished from other groups in the SVM model and the AUC of the AMD, DME, RVO, and CSC groups were 100, 99, 98, and 100%, respectively. While, the AUC of the AMD, DME, RVO, and CSC groups compared with that of other groups in the MLP model only were 99, 97, 97, and 100%, respectively. It could be seen that in terms of ROC curve results, the SVM model has the best performance.

**FIGURE 6:**
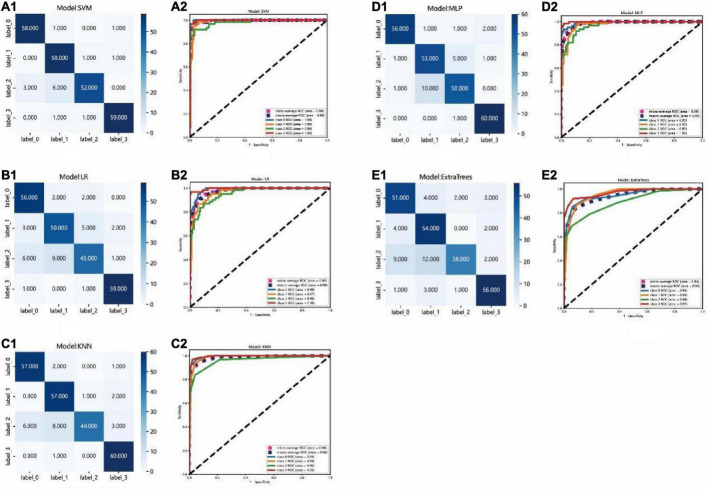
Confusion matrix and ROC curve of the different models: **(A1–E1)**: Confusion matrix of the SVM, LR, KNN, MLP, and ExtraTrees model, respectively. Each row of the matrix represents the actual class and each column indicates the predicted class. **(A2–E2)**: ROC curve of SVM, LR, KNN, MLP, and ExtraTrees model, respectively. Label 0 for AMD, label 1 for DME, label 2 for RVO, label 3 for CSC.

The test set was distributed in a 4 × 4 matrix according to the labeled labels and the classification results. It could be seen that the recognition performance of SVM and MLP models was better than others. For example, the recognition rates of RVO were relatively high in the SVM and MLP model, while RVO was easily misrecognized in the other three models. However, there were also some differences. In the SVM model, the recognition rates of AMD, DME, and RVO were relatively high. While, in the MLP model, the recognition rates of CSC were relatively high. As shown in Figure 6.

## Discussion

The current study used a multi-feature fusion method for automatic ME classification on SD-OCT images. It fused the features of traditional omics and four DL models, which were the alxnet, inception_v3, resnet34, and vgg13. The Grad-CAM was used to visualize the explanation of DL black-box model. Finally, after the fusion features were screened by the lasso model, the non-zero coefficients features were used to developed six classification models. According the accuracy results, as well as the ROC curve and confusion matrix, the performance of the SVM model was the best, and could be used to classify the DME, AMD, RVO, and CSC accurately from DM SD-OCT images.

Early intelligent diagnosis mainly relies on artificially designed feature templates or uses single traditional machine-learning methods (Turchin et al., 2009), treating intelligent diagnosis as a classification problem (Srinivasan et al., 2014; Alsaih et al., 2017). Because a single feature is usually sensitive to the changes of part of the image features and is not sensitive to the changes of other features, when the difference between two kinds of images is not big in the sensitive features of a certain feature, the classifier based on the training of a single feature cannot output the correct classification. In addition, the complex background noise in the image will also lead to the deterioration of feature data quality, which not only increases the difficulty of classifier training, but also reduces the accuracy of classification. Our proposed fusion features method, by contrast, realized feature complementarity and reduced the influence of single feature inherent defects.

In previous studies, Lu et al. (2018) used ResNet to detect normal images, cystoid ME, serous macular detachment, epiretinal membrane, and macular hole based on the single deep learning feature extraction method. The accuracy of their method for detecting cystoid ME cases was 84.8% which was much lower than our result. This also confirmed that the feature fusion method can improve the accuracy of the model compared with the single feature extraction method. Chen T. et al. (2021) used a convolutional-neural-network to classify AMD. Chan et al. (2018) used information from AlexNet, VggNet, and GoogleNet to design a decision model for automatic classification of normal ME and DME. Although these models have performed well, they lack the interpretation capability. The Grad-CAM was introduced in our study to overcome the common drawback of DL models. It uses the gradient of the target class and propagates to the final convolutional layer to generate a rough positioning map, which is used to visualize the features (Yang et al., 2021). The Grad-CAM could address the mechanism by which the CAM approach requires changes to the model architecture. Compared with other interpretation methods, the computational complexity is reduced and the interpretability of the model is increased. It also combines the advantages of fine-grained detection (unable to locate the image) and image positioning (unable to improve the positioning resolution). The result of Grad-CAM heatmaps in our study highlighted important areas that the DL models probably focused on extracting features. This is the same area in which our eyes recognize ME. This is a good example of the Grad-CAM identifying the pathologic region of an OCT image correctly.

Of course, there were also shortcomings to this study. First, we just collected the OCT images from a single-center study so the sample does not represent the entire patient population. Second, single-omics methods were used in this study. For multi-classification, using multi-omics data can obtain better accuracy (Lin et al., 2020). Third, the accuracy of our study needs to be improved. Therefore, in future studies, we will try to incorporate multicenter data to reinforce the conclusion of our study and combined multiomics techniques to automate classification of DM based on SD-OCT images and the color fundus pictures.

## Conclusion

In this study, an artificial intelligence method based on multi-feature fusion was introduced for automatic ME classification on SD-OCT images. The results showed that the model could be used to classify the DME, AMD, RVO, and CSC accurately from SD-OCT images. The result of Grad-CAM heatmaps in our study highlighted important areas that the DL models probably focused on extracting features. The results of Grad-CAM heatmap highlighted that the important areas for the DL model to extract features was the same as the areas in which our eyes recognize ME.

## Data availability statement

The raw data supporting the conclusions of this article will be made available by the authors, without undue reservation.

## Ethics statement

The studies involving human participants were reviewed and approved by the Medical Ethics Committee of the Jiangxi Provincial People’s Hospital. The patients/participants provided their written informed consent to participate in this study. Written informed consent was obtained from the individual(s) for the publication of any potentially identifiable images or data included in this article.

## Author contributions

FG, F-PW, and Y-LZ contributed to data collection, statistical analyses, and wrote the manuscript. All authors read and approved the final manuscript, contributed to the manuscript, and approved the submitted version.
